# Does the implementation of a care pathway for patients with hip or knee osteoarthritis lead to fewer diagnostic imaging and referrals by general practitioners? A pre-post-implementation study of claims data

**DOI:** 10.1186/s12875-019-1044-2

**Published:** 2019-11-09

**Authors:** Esther H. A. van den Bogaart, Mariëlle E. A. L. Kroese, Marieke D. Spreeuwenberg, Ramon P. G. Ottenheijm, Patrick Deckers, Dirk Ruwaard

**Affiliations:** 1 Department of Health Services Research, Care and Public Health Research Institute (CAPHRI), Faculty of Health Medicine and Life Sciences, Maastricht University, Duboisdomein 30, Maastricht, 6229 GT The Netherlands; 2 Research Centre for Technology in Care, Zuyd University of Applied Sciences, Heerlen, The Netherlands; 3 Department of Family Medicine, Care and Public Health Research Institute (CAPHRI), Faculty of Health Medicine and Life Sciences, Maastricht University, Maastricht, The Netherlands; 4 Department of Orthopaedic Surgery, Zuyderland Medical Centre, Sittard-Geleen, the Netherlands

**Keywords:** Care pathway, Stepped care, Osteoarthritis, Diagnostic requests, Referrals, GP, Claims data

## Abstract

**Background:**

The Dutch care for hip and knee osteoarthritis (OA) is of good quality, but there is room for improvement regarding the efficient use of diagnostic imaging and conservative treatment. Therefore a stepped-care approach, in the shape of the care pathway ‘Better exercise in osteoarthritis’, was implemented to reduce the number of diagnostic imaging requested by GPs and referrals of GPs to orthopaedic care.

**Methods:**

In 2015, the pathway is implemented with the use of educational meetings, distributing guidelines and incorporating reminders in the GPs’ referral application. To evaluate the effect of the pathway on the diagnostic and referral behaviour of GPs, hip and knee related health insurance claims are used together with claims of other joints and of a control region for comparison. The average number of claims and the percentage change in the post-implementation period are described. Binary logistic regression analysis is used to examine the interaction between region (intervention and control) and period (pre- and post-implementation). Using random sampling of patient records, information about the practical application of the pathway and the number of hip or knee arthroplasties is added.

**Results:**

In both regions, the number of diagnostic imaging decreased and the number of initial orthopaedic consultations increased during the post-implementation period. Significant interaction effects were found in knee-related diagnostics (*p* ≤ 0.001) and diagnostics of other joints (*p* = 0.039). No significant interaction effects were found in hip-related diagnostics (*p* = 0.060) and in initial orthopaedic consultation claims of hip (*p* = 0.979), knee (*p* = 0.281), and other joints (*p* = 0.464). Being referred according to the pathway had no significant effect on the probability of undergoing arthroplasty.

**Conclusion:**

The implementation of the pathway had a positive effect on GPs diagnostic behaviour related to the knee, but not to the hip. The referral behaviour of GPs to orthopaedic care needs attention for future interventions and research, since an increase (instead of a desired decrease) in the number of initial orthopaedic consultations was found. Focusing on the entire width of care for hip and knee OA could be helpful.

## Background

Osteoarthritis (OA) is a common joint disorder affecting more than half of the population aged 65 years and older [1, 2]. This long-term chronic disease is often associated with stiffness, pain, and functional limitations [3, 4]. Together, this results in a significant reduction in the quality of life of these patients [5, 6].

In 2016, an estimated 1.25 million people (around 7% of the population) had the diagnoses OA in the Netherlands [7]. Annually, there are approximately 140,000 new cases of OA in the country. Knee OA is most common, followed by hip OA. Based on demographic trends, it is expected that the number of people with OA will increase by almost 41% between 2015 and 2040 [7]. Recent increases in the number of people with obesity, a major determinant of OA, suggests that the prevalence of OA is likely to rise in future [8–10]. In 2015, 1.3% of the total cost of health care in the Netherlands was spent on OA-related care [11]. In view of the increasing prevalence, these costs are likely to rise substantially.

In 2014, the Dutch National Health Care Institute (in Dutch: Zorginstituut Nederland) stated that the care for hip and knee OA in the Netherlands is of good quality, but it also emphasized that there is room for improvement in some areas [12]. One of these suggested improvements is related to the efficient use of diagnostic imaging, such as X-ray or magnetic resonance imaging (MRI). OA is primarily a clinical diagnosis [13]. This implies that in most cases the diagnosis can be based on history taking and physical examination [14, 15]. Despite the recommendations in the guidelines [15], Smink et al. [16] found that general practitioners (GPs) often request for diagnostic imaging.

Another suggestion for improvement is related to the treatment of OA. International evidence-based guidelines for hip and knee OA recommend starting with non-surgical (conservative) treatments [17–21]. Joint replacement surgery (arthroplasty) should be performed only in advanced OA and not in the early stages, given the limited lifespan of prostheses and the less successful outcomes of revision arthroplasty [22–24].

Despite the availability of guidelines, several studies have found that a majority of the patients referred to an orthopaedic surgeon did not receive appropriate prior conservative treatment [25–29]. This can be explained by the lack of practical and clear recommendations and strategies about the necessity and sequence of treatment options [30]. A systematic and period approach, a so-called ‘stepped care’ strategy, can be a tool to optimise the use of existing conservative treatment options [31, 32]. Stepped care is characterised by interventions that are offered not earlier or with more intensity than necessary. More radical interventions, like hip or knee arthroplasty, should only be considered when patients do not respond sufficiently to conservative treatment options [27, 33].

An example of a stepped care approach is the care pathway ‘Better exercise in osteoarthritis’ (in Dutch: ‘Beter bewegen bij artrose’) implemented in the Western Mining District of Limburg, in the South of the Netherlands [34]. Various stakeholders, like GPs, physiotherapists, and an orthopaedic surgeon, are involved in this intervention. The pathway is based on the guideline of the Dutch College of General Practitioners (NHG) [35] and aims to treat patients with knee or hip OA according to a stepped care approach. Furthermore, this pathway clearly states that OA is a clinical diagnosis, and therefore X-rays are not necessary.

The pathway may positively influence quality of care and health outcomes. In addition, unnecessary costs could be avoided by implementing these improvements [12]. The Dutch National Health Care Institute estimated that 90%of the costs associated with diagnostic imaging related to both hip and knee OA are unnecessary and that with the implementation of the guidelines, more than €14 million could be saved by deploying conservative treatment [12]. In addition, 5% of hip arthroplasties and 10% of knee arthroplasties could be prevented, based on the assumption that a group of patients is already appropriately managed in primary care. This could result in an additional saving of €34 million [12].

This study aims to evaluate the effect of the implementation of the pathway, on GP diagnostic imaging requests and GP referrals to orthopaedic surgeons for hip and knee OA. In addition, this study evaluates to what extent the pathway is applied in practice before patients are referred to orthopaedic care and the effect of the pathway on the appropriateness of these referrals.

## Methods

### Design

This is an observational study comparing the diagnostic and referral behaviour of GPs in the pre- and post-implementation period of the intervention using health insurance claims data from 2012 to 2016. In addition, a patient record review is conducted to get more information about the practical application of the pathway and the appropriateness of referrals to orthopaedic care within the intervention region.

### Setting

The pathway originates from a regional collaboration of stakeholders in the Western Mining District located in the South of Limburg. Stakeholders consist of a coordination centre for diagnostics, MCC Omnes; the GP organisation Meditta; Zuyderland Medical Centre (MC) (location Sittard-Geleen); the health insurance company CZ; and a patient representative organisation, Citizen Power (in Dutch: Burgerkracht). These organisations work together to provide the right care in the right place [34].

The Western Mining District has a population of about 185,000 people. The population is declining and ageing [36]. The control group incorporated three other regions. These regions are selected because they are located in the same province as the intervention region and are also characterized by a declining and ageing population. Together, these control regions have a population of about 690,000 people.

In the Netherlands, having health insurance is mandatory [37]. In the Western Mining District, CZ is the health insurance company with the largest market share in the region. In addition, all Dutch residents are registered at a GP practice. Primary care is delivered by GPs, which initiates diagnostics and acts as a gatekeeper to specialised medical care [38]. GP consultations are fully covered by the health insurance [39]. For consulting a medical specialist, a yearly compulsory deductible is levied. This implies that there is a certain amount of specialised medical treatment expenses that a patient has to pay out of pocket before the health insurance company will compensate the expenses. The same applies for diagnostic tests (including diagnostic imaging) and pharmaceuticals prescribed by GPs. The amount of the deductible is determined by the Dutch government and changes every year [40]. During the study period (2012–2016), the amount increased from €220 to €385.

### Intervention

The pathway is designed, using the national guidelines for hip and knee OA [34], by members of an expert group, consisting of two GPs, a physical therapist, an orthopaedic surgeon, a rheumatologist, a radiologist, a physician assistant and a coordinator of MCC Omnes. In February 2015, the pathway is implemented in the Western Mining District based on three interventions: educational meetings, distribution of the guidelines, and reminders. All interventions are developed and coordinated by members of the expert group and focus on improving the stepped care approach of hip and knee OA treatment, reducing diagnostic imaging requests and reducing referrals to orthopaedic surgeons. The educational meetings consist of one meeting organised for GPs and physiotherapists together at the start of the implementation process, followed by advanced educational courses organised separately for GPs and physiotherapists. The educational meetings focus on recognizing OA and red flag situations, the content of the pathway and related guidelines, the role of different professionals within the pathway (with emphasis on GPs and physiotherapists) and the application of the pathway in practice, for example by discussing practical cases, patient communication skills training and practicing administering corticosteroid injections. The educational meetings are voluntary and professionals earn medical education credits for their presence. Around 20% of the GPs in the intervention region attended the first education meeting. In addition, the expert group assumes that the attended GPs spread the content of the meetings among their colleagues within their general practice and that all GPs, affiliated with MCC Omnes, eventually conform to their initiatives. To further support the dissemination of the pathway, a visualisation and explanation of the stepped-care approach are placed at the website of MCC Omnes, and in the newsletter and on the mobile application of MCC Omnes to reach all GPs in the region. In addition, to support GPs in applying the pathway in practice, a reminder pops up in the GPs’ referral application (called ZorgDomein) when requesting hip or knee related diagnostic imaging or when referring patients with hip or knee related complaints to orthopaedic care. This reminder forces GPs to indicate which steps of the pathway have been followed prior to the request or referral. Hence, all GPs are informed about the pathway through these different channels. The pathway focusses on patients with hip and knee OA diagnosed by their GP based on history taking and physical examination. In cases of rheumatic diseases, previous diagnosis of OA, OA that cannot be explained sufficiently, young age (< 45 years), prominent polyarthritis (in multiple joints), (familial) psoriasis, or inflammatory bowel disease, patients are excluded from being treated according to the pathway.

**Fig. 1:**
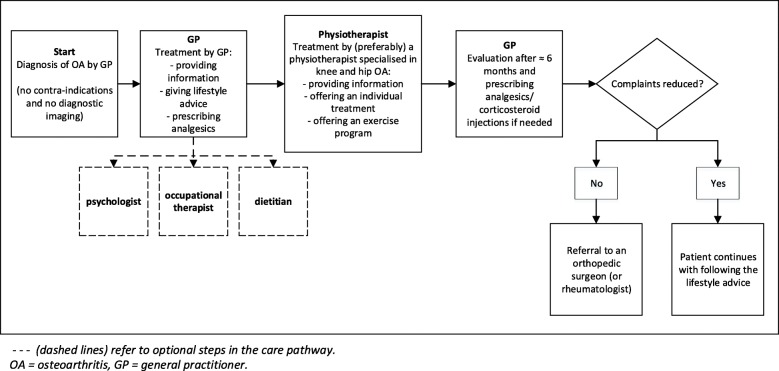
Process of the stepped care approach in the pathway

Figure 1 shows the stepped care process of the pathway. When patients are diagnosed with OA by their GP and patients are eligible for treatment according to the pathway, the GP provides information about OA and advice about lifestyle. When necessary, analgesics are prescribed. The GP refers the patient, according to the conservative policy, to a physiotherapist and when necessary to a dietitian, psychologist (in case of problems with coping), and/or occupational therapist. The physiotherapist provides more (tailored) information about OA to the patient. In addition, an individual treatment process is started, aimed at reducing pain and functional disorders, combined with an exercise program focussing on guiding patients to a more active lifestyle. Preferably, after approximately 6 months, the GP evaluates the results of the conservative treatment. When complaints reduce, patients are advised to continue with the lifestyle advice. When complaints not reduce, additional analgesics could be prescribed or corticosteroid injections are administered. Finally, patients could be referred to specialised medical care (mostly a referral to an orthopaedic surgeon).

### Data collection

#### Observational study

The effect of the pathway is assessed using annual health insurance claims data (2012 to 2016) from the health insurance company CZ. Claims data can be categorized as health care administrative data [41]. The health insurance company collects these data for administrative and billing purposes, and they can be used to study health care consumption. The claims data are based on measurements for all CZ insured persons within the intervention and control region (e.g. census data).

The number of hip- and knee-related claims are compared with claims related to other joints (neck, shoulder, upper arm, elbow, forearm, hand, wrist, fingers, ankle, foot, and toes). Additionally, the number of health insurance claims in the Western Mining District (intervention region) are compared with the number of claims in the control region.

#### Patient record review

To collect information about the practical application of the pathway and the appropriateness of referrals to orthopaedic care within the intervention region, a patient record review is conducted using random sampling. Approximately 10% of the total number of the records of patients with a suspicion of hip or knee OA, who were referred for an initial orthopaedic consultation during the period from February 2015 to October 2016 (post-implementation period), are included.

### Outcome measures

#### Observational study

Regarding the claims data, the primary outcome measures are the number of hip- and knee-related diagnostic imaging procedures (X-rays and MRIs) requested by GPs and the number of GP referrals to orthopaedic care per 1000 insured persons.

Based on the claims data, it is not possible to determine the total number of GP referrals to orthopaedic care, due to a lack of follow-up of the referral or non-attendance [42]. Therefore, the actual claims of initial orthopaedic consultations related to OA of the hip and knee and other joints are used, both in the intervention and control region. Initial consultations are recognized by the so-called Diagnosis Treatment Combination (DTC) (in Dutch: Diagnose Behandel Combinatie, DBC) [43]. Every DTC has a unique performance code that includes all information about the type of care (initial or follow-up), the demand for care, the diagnosis and type of treatment.

The characteristics of the entire population insured by CZ in the intervention region and the control region are 1) the number of insured persons, presented with averages per period, 2) gender distribution, presented using the percentages of men, and 3) average age, presented with means and standard deviations (SDs).

The pre- and post-implementation period is determined by the implementation of the pathway in the beginning of 2015. Therefore, the pre-implementation period is determined as January 2012 to December 2014 and the post-implementation period is determined as January 2015 to December 2016. A post-implementation period of 2 yrs is selected. It is not possible to select a period of 3 yrs due to the delay in the processing of the claims data by the health insurer.

#### Patient record review

The collected patient records using the random sampling contain the answers to the questions related to the reminder that pops-up in the GPs’ referral application when they refer patients to orthopaedic care (“Did you (the GP) went through the steps of the pathway?”). The answers to this question (yes or no) are used to check whether patients had been referred according to the pathway. Furthermore, the patients’ records contain information about the follow-up orthopaedic care in the hospital. Information about the diagnosis (yes/no OA) and the treatment (yes/no arthroplasty) are collected from these records to check if the referral was appropriate.

### Analysis

#### Observational study

Characteristics of the intervention and control region during the pre- and post-implementation period are presented. In addition, the average number of health insurance claims for diagnostic imaging and initial orthopaedic consultations (separately for hip, knee, and other joints) per 1000 insured persons in the pre- and post-implementation period of both regions are reported. The percentage change in the number of requested diagnostic imaging and initial orthopaedic consultations in the post-implementation period compared to the pre-implementation period is calculated and presented. Furthermore, the proportion of diagnostic imaging and initial orthopaedic consultations claims per 1000 insured persons per region and per period are dichotomised to a binary variable (yes/no-claimed diagnostic imaging or initial orthopaedic consultation).

Binary logistic regression analyses are used to determine the influence of the implementation of the pathway on the proportion of health insurance claims for diagnostic imaging and initial orthopaedic consultations per 1000 insured persons in the intervention region compared to the control region. The dependent variable in these models is the binary variable indicating claimed diagnostic imaging (yes/no) or claimed initial orthopaedic consultation (yes/no). The independent variables are region (intervention or control region) and period (pre- or post-implementation period) and the interactions between those variables. The Enter method is used [44]. In addition, odds ratios (OR), *p*-values, and 95% confidence intervals (CI) are reported.

#### Patient record review

The number and percentage of patients referred to orthopaedic care according to the pathway, the number and percentage of patients diagnosed with OA as determined by the orthopaedic surgeon and the number and percentage of arthroplasties performed are presented in flow charts, separately for patients with a suspicion of hip and knee OA. Pearson’s chi-square test is used to test the difference in the probability of undergoing arthroplasty between patients who were referred according to the pathway and patients who were not, again separately for patients with hip and knee OA.

Descriptive statistics and analyses are performed using SPSS version 25, and statistical significance is defined as *p* < 0.05 (IBM SPSS Statistics, Armonk, NY).

#### Expert meetings

The process of the pathway and results of the analyses are discussed during meetings with the expert group. The purpose of these meeting is to verify the findings and to contribute to a better interpretation of the results.

## Results

### Observational study

Table 1 shows that the number of insured persons decreased over time in the intervention region. In the control region, the number of insured persons increased. However, the proportions of men and the mean age remained stable over time in both regions. Therefore, it is assumed that the effect of the decrease in the intervention region is limited.

**Table 1:** Characteristics of intervention and control region

Region and period	Average number of insured persons (N)	Gender – male (%)	Age in years (mean ± SD)
Intervention region			
Pre	85,749	48.3	45.92 ± 23.26
Post	80,078	48.4	46.36 ± 23.40
Control region			
Pre	295,796	48.9	45.32 ± 23.26
Post	299,306	48.8	45.78 ± 23.39

The number of claims and the percentage change in the intervention and control region are described (Table 2). In both regions, the average number of requested diagnostic imaging procedures decreased during the post-implementation period. Regarding the initial orthopaedic consultations, an increase of claims during the post-implementation period in both regions is visible.

**Table 2:** Number of claims per region in the pre- and post-implementation period

	Requested diagnostic imaging			Initial orthopaedic consultation		
	Pre	Post		Pre	Post	
	Average per 1000 insured persons (N)	Average per 1000 insured persons (N)	Percentage change (%) *	Average per 1000 insured persons (N)	Average per 1000 insured persons (N)	Percentage change (%) *
Intervention region						
Hip-related	15.19	13.45	– 11.5	10.92	13.14	+ 20.3
Knee-related	12.72	9.51	– 25.2	5.93	6.47	+ 9.1
Other joints	35.45	32.14	– 9.3	3.07	3.60	+ 17.3
Control region						
Hip-related	15.94	15.43	– 3.2	11.30	13.50	+ 19.5
Knee-related	13.24	12.91	– 2.5	5.37	6.77	+ 26.1
Other joints	43.95	43.73	– 0.5	5.04	5.34	+ 6.0

* – = percentage change is negative (a decrease), and + = percentage change is positive (an increase)

As presented in Table 3, there was no statistically significant difference in the decrease of the number of GP-requested hip-related diagnostic imaging during the post-implementation period (OR = 0.903, 95% CI = 0.812–1.004, *p* = 0.060) in the intervention region compared to the control region. However, during the post-implementation period the number of GP-requested knee-related diagnostic imaging (OR = 0.781, 95% CI = 0.693–0.880, *p* ≤ 0.001) and GP-requested diagnostic imaging of other joints (OR = 0.931, 95% CI = 0.870–0.997, *p* = 0.039) declined statistically significantly more in the intervention region.

**Table 3:** Results of the logistic regression analysis for claimed diagnostic imaging

	Odds Ratio	P-value	95% Confidence Interval	
			Lower Bound	Upper Bound
*Hip-related*				
Period	0.981	0.573	0.916	1.050
Region	0.895	0.003 *	0.832	0.963
Region x period	0.903	0.060	0.812	1.004
*Knee-related*				
Period	0.955	0.225	0.886	1.029
Region	0.898	0.008 *	0.829	0.973
Region x period	0.781	0.000 *	0.693	0.880
*Other joints*				
Period	0.970	0.152	0.930	1.011
Region	0.764	0.000 *	0.729	0.801
Region x period	0.931	0.039 *	0.870	0.997

* p  < 0.05

Moreover, there was no statistically significant difference in the increase of the number of initial orthopaedic consultation claims of the hip (OR = 1.002, 95% CI = 0.871–1.152, p = 0.979), knee (OR = 0.894, 95% CI = 0.728–1.097, p = 0.281), or other joints (OR = 1.091, 95% CI = 0.864–1.378, p = 0.464) in the intervention region compared to the control region (Table 4).

**Table 4:** Results of the logistic regression analysis for initial orthopaedic consultation claims

	Odds Ratio	P-value	95% Confidence Interval	
			Lower Bound	Upper Bound
*Hip-related*				
Period	1.103	0.039 *	1.005	1.209
Region	0.939	0.222	0.849	1.039
Region x period	1.002	0.979	0.871	1.152
*Knee-related*				
Period	1.112	0.128	0.970	1.274
Region	1.015	0.844	0.877	1.173
Region x period	0.894	0.281	0.728	1.097
*Other joints*				
Period	1.007	0.922	0.878	1.155
Region	0.600	0.000 *	0.509	0.709
Region x period	1.091	0.464	0.864	1.378

* p  < 0.05

### Patient record review

**Fig. 2:**
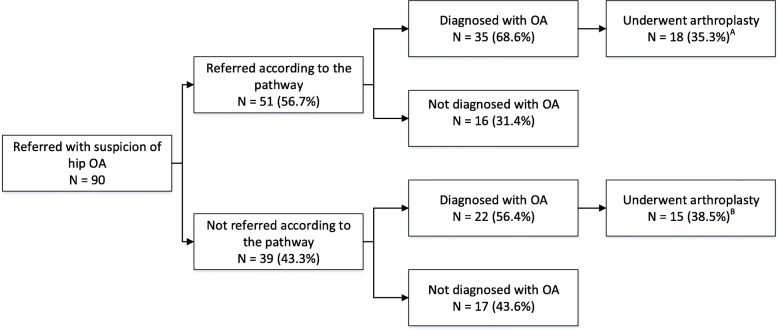
Flow chart of the probability of arthroplasty for patients referred with suspicion of hip OA ^*A*^ *Percentages are calculated based on the number of patients referred according to the pathway (N = 51)* ^*B*^ *Percentages are calculated based on the number of patients not referred according to the pathway (N = 39)*

**Fig. 3:**
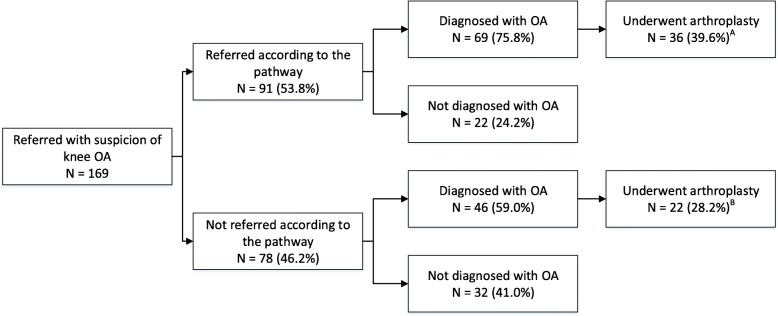
Flow chart of the probability of arthroplasty for patients referred with suspicion of knee OA ^*A*^ *Percentages are calculated based on the number of patients referred according to the pathway (N = 91)* ^*B*^ *Percentages are calculated based on the number of patients not referred according to the pathway (N = 78)*

Figures 2 and 3 show the number and percentage of patients referred by their GP (from the intervention region) to orthopaedic care with a suspicion of hip or knee OA and the number of performed arthroplasties. Figure 2 shows that the majority of patients with a suspicion of hip OA (56.7%) were referred according to the pathway. After the referral, 68.6% of the patients who were referred according to the pathway were diagnosed with OA. For patients not referred according to the pathway, this percentage was 56.4%. Finally, the percentage of patients who underwent arthroplasty was lower for patients referred according to the pathway than for patients not referred according to the pathway (35.3 and 38.5%, respectively). In addition, Fig. 3 shows that the majority of patients with a suspicion of knee OA (53.8%) were referred according to the pathway. After the referral, 75.8% of the patients who were referred according to the pathway were diagnosed with OA. For patients not referred according to the pathway, this percentage was 59.0%. Finally, the percentage of patients who underwent arthroplasty was higher for patients referred according to the pathway than for patients not referred according to the pathway (39.6 and 28.2%, respectively).

Table 5 shows that for both patients with a suspicion of hip and knee OA, the probability of undergoing arthroplasty does not significantly differ for patients who were or were not referred according to the pathway.

**Table 5:** Percentages and numbers of arthroplasty in patients being referred or not according to the pathway

	Total N	Arthroplasty % (N)		*P*-value ^a^
		Yes	No	
Hip OA	90			0.757
Referred according to the pathway	51	35.3 (18)	64.7 (33)	
Not referred according to the pathway	39	38.5 (15)	61.5 (24)	
Knee OA	169			0.121
Referred according to the pathway	91	39.6 (36)	60.4 (55)	
Not referred according to the pathway	78	28.2 (22)	71.8 (56)	

^*a*^ *Tested with Pearson’s chi-square test*

## Discussion

The present study found a significant decrease during the post-implementation period in the number of knee-related diagnostic claims per 1000 insured persons in the intervention region compared to the control region. A similar decrease in the number of requested diagnostic imaging procedures for other joints was found. This decrease indicates that the implementation of the pathway went beyond awareness of requesting hip- and knee-related diagnostic imaging by GPs and positively influenced the GPs when it came to requesting diagnostic imaging in general. No differences in the number of hip-related diagnostic imaging procedures was found between the intervention and control region during the post-implementation period. Additionally, the pathway seems to have less effect on the referral behaviour of GPs. Regarding to the claims data, a significant difference in the number of initial orthopaedic consultations in the intervention region during the post-implementation period compared to the control region was not found. Furthermore, the random sample of the patient records showed that not all GPs seem to conform to the pathway since almost half of the patients were not referred to orthopaedic care according to the pathway. Besides this, patients with a suspicion of hip or knee OA referred to orthopaedic care according to the pathway had the same probability to undergo arthroplasty than patients referred not according to the pathway. This seems to indicate that the quality of referring did not improve through the implementation of the pathway.

The results of the present study regarding the absence of a significant decrease in the number of hip related diagnostic imaging are in line with the study of Linsell et al. [45] in which GPs were more likely to request an x-ray for older people with hip pain than for older people with knee pain. A possible explanation for this could be the fact that hip complaints are more complex for GPs to manage. Literature shows that pain from the hip is difficult to define and that it is difficult to determine the exact source of pain [46, 47], that hip OA patients have more advanced complaints and that triggers for symptomatic presentation in hip OA are less understood [48]. When GPs experience difficulties in diagnosing hip related complaints, requesting diagnostic imaging can be a strategy to deal with these uncertainties [49]. Therefore, improving GPs skills to set the diagnosis OA of the hip could be the focus in future educational meetings. In addition, during the expert meetings, GPs revealed that it could be difficult to convince patients that diagnostic imaging is not always necessary to diagnose OA. Previous studies [50–52] have found that GPs’ perception of patient pressure influences the non-adherence to guidelines concerning indications for diagnostic imaging, like an X-ray or MRI. Moreover, Baker et al. (2006) found that GPs believed that denying an X-ray could adversely affect the doctor–patient relationship. Although patient communication was part of the educational meetings, further improving GPs’ patient-centred communication skills can be useful, since these skills are associated with fewer diagnostic testing expenditures [53].

An explanation for the lack of effect found in the present study regarding the referral behaviour of GPs may be the fact that the practical application of the pathway is not optimal since not all patients were referred to orthopaedic care according to the pathway. This could explain why no decreasing effect was found in the number of initial orthopaedic consultation claims. According to Rogers [54], when implementing an innovation, a part of the target group is sceptical and will offer resistance to change behaviour. Therefore, gaining insight into the application of the pathway by GPs may provide valuable information about the non-users. These insights can be used to evaluate barriers for application and to tailor interventions in order to stimulate the practical application [55]. Another explanation for the lack of effect could be the worldwide consistent increase in the incidence of joint arthroplasty [56]. This also explains the similar increase found in the control region. Additionally, during the expert meetings it emerged that patients can have a strong preference for a referral to orthopaedic care. Therefore, a referral sometimes is the only way to let patients accept that surgical treatment might not be beneficial. Literature confirms that patients’ preferences and GPs’ perception of patient pressure indeed influence the GP referral behaviour [52, 57]. This supports the evidence that guidelines are relatively ineffective when implemented on their own [58–60]. Again, improving patient-centred communication skills can be useful. Furthermore, increasing the consultation time per patient was mentioned during the expert meetings as an important criterion to apply these skills properly. Literature shows that longer consultations are associated with greater patient enablement [61], higher patient-centeredness [62], and a higher degree of offering lifestyle advice and preventive activities [63]. However, evidence about the influence of consultation length on the number of referrals and patient satisfaction is lacking [64]. In addition, exploring other interventions focussing on referring more appropriately to specialised medical care can be beneficial to reduce the inefficient use of limited resources [65–67]. Examples of such interventions are peer-reviewing referrals within a general practice before sending them to specialised medical care, enabling GPs to obtain the advice of medical specialists, periodic visits by medical specialists to GP practices, and shifts to outpatient clinics in which orthopaedic surgeons or other health-care professionals with a special interest in musculoskeletal problems (for example GPs, nurse practitioners, or physician assistants) provide care in a community setting [67]. These alternatives appear promising with respect to reducing unnecessary referrals to specialised medical care but require further investigation into the effects on quality of care, patients’ experiences, and cost of care [67]*.*

Another point of attention mentioned during the expert meetings was the quality of physical therapy. Although the educational meetings and the distribution of the guidelines also aimed at physiotherapists, the focus of this study was on the impact of the implementation of the pathway on GPs behaviour. However, if physiotherapist do not use conservative treatment optimally and patients therefore do not experience improvements, GPs may feel forced to refer patients to orthopaedic care. Therefore, the practical implications of the pathway for physiotherapists and possibly other healthcare professionals (such as dieticians and psychologists) should be addressed in future research.

Based on the results, it is difficult to indicate which intervention (educational meetings, distribution of the guidelines, or reminders) contributed most to the decrease in the number of requested diagnostic imaging procedures related to the knee and other joints since this study did not examined the effect of the different interventions separately. However, in a systematic review conducted by French et al. [68] reminders were mentioned as potentially effective to change health professional behaviour and improve the use of diagnostic imaging. In the same review, educational meetings were not shown to be effective for changing imaging ordering behaviour. Furthermore, Hollingworth et al. [69] found no evidence about the effect of distributing clinical guidelines on changing GPs imaging behaviour related to patients with lumbar spine complaints. In addition, according to the literature [70], the educational meetings organised by members of the expert group have potential to impact on referral rates. More research is needed to learn about the effects of the various interventions within the context of this pathway. This information is needed to further optimise the implementation of the pathway and to achieve a further increase in the appropriate use of diagnostic imaging and possibly achieve a decrease in the number of referrals to orthopaedic care.

## Limitations

In the present study, annual claims data were used. To ensure anonymity, only aggregated data (number of claims per year) were available and no analysis at an individual level could be made. Furthermore, claims data from only one health insurance company were used, which limits the ability to generalise the results of the study to a wider population (external validity) [71]. Since data from the health insurance company with the largest market share in the region were used, problems of selection bias are limited.

In addition, data on the exact extent to which the pathway was applied at GP level in the intervention region were missing. Although all GPs were informed about the pathway, information about how many GPs requested diagnostic imaging procedures and referred patients to orthopaedic care according to the guidelines was lacking. This makes it difficult to attribute the implementation results of the pathway and to consider if there is more room for improvement. Therefore, more research is needed to measure the “real” effect of the pathway in the future.

Furthermore, the present study focused merely on the effects of the implementation of the pathway on GPs behaviour. However, healthcare professionals like physiotherapists, dieticians and psychologist are also involved in the conservative treatment of patients with hip and knee OA. Therefore, research on the effects of the pathway across the entire width of care for hip and knee OA is necessary in order to improve the effect of the pathway.

Finally, it is important to focus not only on the number of requested diagnostic imaging procedures and referrals to orthopaedic care, but also on the effect of the pathway on patient satisfaction, quality and costs of care [72]. Therefore, more extensive research in patients, for example through the use of questionnaires, is needed [73].

## Conclusion

The introduction of a pathway aiming to reduce GP diagnostic imaging requests and GP referrals to orthopaedic surgeons for hip and knee OA, had mixed effects. Results showed a decrease in the number of diagnostic imaging requests for knee and other joint related OA, but no impact was found on those for hip OA. In parallel, referrals to orthopaedic care increased during the post-implementation period, both for hip and knee OA related referrals.

Future research is needed to identify the specific role of the interventions in their effectiveness in improving the diagnostic behaviour of GPs, particularly related to diagnostic imaging procedures of the hip. In addition, further research on the referral behaviour of GPs is necessary, which should focus on possible other interventions and the entire width of care for hip, and knee OA in order to improve the effect of the pathway.

## Abbreviations

CI: Confidence interval
GP: General Practitioner
MC: Medical centre
MRI: Magnetic resonance imaging
NHG: Dutch College of General Practitioners (in Dutch: Nederlands Huisartsen Genootschap)
OA: Osteoarthritis
OR: Odds ratio
SD: Standard deviation
